# Acute Myeloid Leukemia: Is That All There Is?

**DOI:** 10.7759/cureus.3198

**Published:** 2018-08-24

**Authors:** Aneeqa Saif, Syed Faheem Ali Kazmi, Rabia Naseem, Haider Shah, Mohammad Omar Butt

**Affiliations:** 1 Sindh Medical College, Dow University of Health Sciences, Karachi, PAK; 2 Medicine, Dow University of Health Sciences, Karachi, PAK

**Keywords:** acute myeloid leukemia, leukemia, acute disease, cell transformation, neoplasia, genetics, chromosomal aberrations, chromosomal classification, cytogenetic analysis

## Abstract

Acute myeloid leukemia (AML) is characterized by the clonal proliferation of malignant myeloid blast cells in the marrow along with impaired normal hematopoiesis. With an almost stagnant approach for the management of patients with AML in the last three decades, the main purpose of this paper is to increase our understanding of recent scientific advancements for the enhanced diagnosis and treatment of AML. Existing research data related to different approaches for a possible improvement in AML management has been collected and discussed. The identification of recurrently mutated genes, such as CCAAT-enhancer-binding proteins α (CEBPα), Fms-related tyrosine kinase 3 (FLT3), and nucleophosmin 1 (NPM1) along with the classic diagnostic karyotype has improved prognostic-risk stratification. Moreover, mutations affecting cellular metabolism like isocitrate dehydrogenase (IDH1), lysine-specific demethylase 1 (LSD 1), and nuclear factor kappa-light-chain-enhancer of activated B cells (NF-κB) have become a huge success by providing targets for novel therapeutic drugs. Checkpoint inhibitors (CPI) and vaccination against tumor-associated antigen are added options considered, which require further trials before their efficacy can be determined. An important tool in monitoring early response to therapy, minimal residual disease (MRD) assays can be further refined by including pretreatment parameters such as cytogenetic and molecular markers. Potential side effects and resistance to treatment remains a huge barrier in completely finding success against AML and work needs to be done to find combinations of immunotherapies to possibly reduce adaptive resistance by AML.

## Introduction and background

Acute myeloid leukemia (AML) represents a heterogeneous and malignant clonal disorder of the hematopoietic system, characterized by uncontrolled proliferation, a lack of differentiation of immature, abnormal blast cells, in addition to the impaired production of normal blood cells. While significant progress has been achieved in the scientific understanding of AML, the treatment has not changed meaningfully in the last three decades [1].

The focus of this manuscript is to discuss major areas of recent scientific advancement and provide expectations towards the improved diagnosis and treatment of AML in the near future.

## Review

Epigenetic regulation 

One of the recent successes in AML research is understanding the role of epigenetic dysfunction in its pathogenesis. Recurrent mutations affecting cellular metabolism are implicated in the pathogenesis. Although heterogeneous in nature, mutations in genes encoding epigenetic regulators is a common occurrence in AML. For instance, while the prognostic significance of the mutually exclusive isocitrate dehydrogenase (IDH) 1 and 2 mutations is, to date, uncertain, collectively, various mutations of these two genes have been found to be present in AML [2-3]. These prove to be a novel target for the development of leukemia-specific therapies with various studies demonstrating granulocytic differentiation at the level of leukemic blasts and more immature stem-like cells in vitro by the utilization of IDH 1/2 inhibitors [4-5]. Likewise, the histone demethylase, lysine-specific demethylase 1 (LSD1), has emerged as a promising therapeutic target in multiple cancers, notably in AML with a reportedly improved survival of mice engrafted with human AML cells in response to a combination of an LSD1 antagonist and a pan-histone deacetylase inhibitor [6]. However, various reversible side effects have been reported by the loss of LSD1, which include severe anemia and impaired erythropoiesis [7]. Moreover, decitabine, a hypomethylating agent, demonstrated improved response rates in older patients with AML compared to standard therapies in a phase three trial [8], similar to the methyltransferase inhibitor azacytidine, which demonstrated increased survival in patients with intermediate-2 and high-risk myelodysplastic syndromes and is widely used [9].

There is a need for cooperative groups or larger studies to improve our knowledge of the more rare mutations and the characteristics of patients with certain co-occurring mutations. For instance, Ley et al. reported the presence of at least one potential driver mutation in nearly all AML samples and nine functionally related categories of mutated genes involved in the pathogenesis of AML, describing a unique relationship of collaboration and mutual exclusivity among them [10]. Another study demonstrated improved outcomes in patients with DNMT3A {deoxyribonucleic acid (cytosine-5)-methyltransferase 3A} and nucleophosmin 1 (NPM1) mutations and mixed lineage leukemia (MLL) translocations in response to high-dose induction chemotherapy. Hence, apart from the beneﬁt of achieving a greater consensus in risk stratification, a mutational analysis could also further aid in therapeutic decisions by recognizing newer subgroups, such as a subgroup that shows better results with induction and consolidation therapy while another subgroup with mutationally defined unfavorable outcomes would prove to be a potential applicant for allogeneic stem-cell transplantation [11].

One drawback of cancer immune therapy for hematological malignancies is that the effector immune cells may potentially be malignant themselves. Somewhat paradoxically for an apparently immune-responsive malignancy, there is mounting evidence that AML is an immunosuppressive or at least immunoevasive disease [12].

Le Dieu and coworkers identified higher amounts of clusters of differentiation 3+ (CD3) T cells and CD8+ T cells in the peripheral blood (PB) of AML patients as well as a higher expression of activation markers, such as CD25 and CD69, concurrent with a higher expression of memory markers, thus concluding that in AML, the immune system is in a primed and activated state [13].

Several novel cancer immune therapeutic treatment options for the hematological malignancies are entering the clinic. Additionally, a plethora of novel and highly innovative treatment modalities are currently being tested in both the clinical and preclinical settings. Progress has been shown in treatments for the lymphoid malignancies where, above all, chimeric antigen receptor (CAR) T cells and bispecific T cell engagers (BiTEs) have demonstrated remarkable clinical effects [14].

Checkpoint inhibitors (CPI) as positive modulators of immune response have changed the therapeutic landscape of several solid malignancies dramatically [15]. Accordingly, checkpoint inhibitors have also been tested in patients with AML, albeit the results have only been reported as abstracts and there are no robust clinical data or large randomized clinical trials. All available data are derived from preclinical studies or extrapolated from phase I/II trials; CPI had a modest clinical efficacy as a single agent, and It seems to be more active in combination with other agents, e.g. hypomethylating agents (HMA) or combination CPIs [16].

Vaccination

Given the fact that several tumor-associated antigens (TAAs), such as Wilms tumor antigen 1 (WT1), NY-ESO1, PR1 (a nonamer epitope derived from neutrophil elastase and proteinase 3), and PRAME (preferentially expressed antigen of melanoma) are highly expressed in AML, several trials have tested vaccines [17]. Vaccines are well-tolerated but likely to be effective only in patients in apparent remission since they do not induce a potent T cell response. An interesting aspect in the treatment of AML is the prospect of combining HMA with peptide vaccinations, as it has been shown that HMA enhances the expression of TAA, most notably NY-ESO as well as increases the amount of cancer germline antigen (CGA)-specific CD8+ T cells [18]. Speculations in the immunogenic potential of the neo-antigens generated by the Fms-related tyrosine kinase 3 (FLT3), internal-tandem-duplicate (ITD) mutations, which confer a dismal prognosis, have also been discussed. However, vaccines are likely to be more effective in patients who achieve a complete remission after prior chemotherapy [16]. The dysregulation of transcription factors caused by gene mutations, chromosomal aberrations, or aberrant expression can lead to cancer, including acute myeloid leukemia [19].

The recognition of FLT3 as one of the genes commonly undergoing mutation in AML has allowed for a thorough exploration as a target for therapy. FLT3 inhibitors have been shown to induce antineoplastic activity in patients with relapsed or refractory AML, especially with those patients shown to have FLT3 mutations. Indeed, highly potent FLT3 inhibitors, such as quizartinib (AC220), have generated substantial antileukemic clinical effects among relapsed/refractory AML patients with FLT3-ITD mutations, as well as a smaller number of patients without a documented FLT3 mutation. The inhibition of FLT3 was believed to be via the induction of direct cytotoxicity in the myeloblasts. However, there is evidence to suggest that in a subset of the population responding to FLT3 inhibitors, the response is not through direct cytotoxicity but via a terminal differentiation of blasts in patients with FLT3-ITD mutations [19].

Minimal residual disease monitoring (down to the last leukemia stem cell)

Minimal residual disease (MRD) monitoring provides a potential tool to not only evaluate the early response to therapy but to further decide post-remission strategies. It has become more difficult to assess in AML due to the large number of recurrent mutations, molecular heterogeneity of pre-leukemic and leukemic clones, and clonal genetic instability. The most widely used methods to establish MRD include polymerase chain reaction (PCR) and multiparametric flow cytometry. In APL, MRD has been known to identify the majority of patients subjected to relapse and proven to be the most potent predictor of relapse-free survival [20]. Likewise, in AML, it is imperative to establish standardized MRD assays and validated prognostic MRD thresholds, a goal hindered greatly by small patient numbers and the lack of availability of serial samples at distinct time points [20].

MRD does not always reflect the total leukemic burden and the persistence of residual leukemia stem cells (LSC) may explain why a certain proportion of MRD-negative patients experience disease recurrence. The combination of putative LSC frequency and MRD frequency yields four patient groups with different survivals: good, intermediate, poor, and very poor patients. Hence, amalgamating our knowledge of the residual LSCs and the “whole MRD” fractions may improve prognostic information at follow-up, presenting an added method to further improve assessment and guide future therapeutic interventions [21]. By using techniques such as whole-genome or exome sequencing and targeted deep sequencing, Klco et al. demonstrated a significantly increased risk of relapse and a decrease in overall survival in at least 5% of bone marrow cells that had persistent leukemia-associated mutations in day-30 remission samples, highlighting the significance of the genomic approach in the improvement of risk stratification for patients with AML [22].

Compared with cytotoxic chemotherapy, epigenetically active agents and targeted small molecule inhibitors, immunotherapy approaches have non-overlapping toxicity and efficacy profiles. Thus, future directions should combine the various tools that have been developed against AML in the last few years.

In addition, we can sequence samples over time to learn about tumor heterogeneity to predict changes in tumor phenotype and treat them accordingly. In the future, each tumor subtype may be sequenced with such patients being followed longitudinally to keep track of the molecular changes. This could help in identifying a finite number of clonal and sub-clonal mutations and further enable us to recognize the signaling pathways involved. Keeping in perspective tumor heterogeneity and single-cell genome sequencing in addition to highlighting co-occurring or mutually exclusive mutations also facilitates the approximation of frequency of individual mutant alleles in cancer with one study relating the cytotoxic response to selective FLT3 inhibition with a high allelic mutant burden in an FLT3/ITD specimen [23-24].

The role of the leukemia microenvironment

Interaction between leukemia cells and bone marrow mesenchymal stromal cells (BM-SC), a key component of the bone marrow microenvironment, initiates a complex network of tumor-promoting factors that include transcription factors, microribonucleic acid (miRNA), adhesion and signaling molecules, cytokines and chemokines, which results in the development of stroma-mediated chemo-resistance aiding in the survival and proliferation of leukemia cells. The role of NF-κB (nuclear factor kappa-light-chain-enhancer of activated B cells) activation has also been suggested in the development of resistance to anti-leukemic drugs and various NF-κB inhibitors have shown to successfully eradicate cancer stems cells; however, the inhibition of NF-κB alone is not sufficient to eradicate the AML stem cells [25]. Similar results have been obtained by targeting biomarkers, such as BCL2 (B-cell lymphoma 2), CD123, or the mTOR (mammalian target of Rapamycin) pathway, paving way for a continued search for other such possible targets [26-28].

Other than the leukemia-stroma connection, hypoxia is another microenvironmental factor that may affect the response of cells to chemotherapy. Benito et al. demonstrated prolonged survival and decreased leukemia burden as a result of the administration of the hypoxia-activated dinitrobenzamide mustard, PR-104, in immunodeficient mice injected with primary acute lymphoblastic leukemia cells [29]. Patient-derived xenografts (PDX) in mice manage to express some of the heterogeneity by preserving the clone mutations in the PDX cells; however, sub-clonal architecture is often not reflecting the primary sample creating a need for xenotransplantation models to be controlled by characterizing the genotype of AML cells both before and after xenotransplantation [30-31]. Carter et al. studied leukemic cells co-cultured with BM-SCs under hypoxic conditions and suggested the utilization of extrinsic pathways to eradicate leukemia stem cells that are resistant to therapy, by the administration of a combination of an endogenous antagonist of inhibitors of apoptosis (IAP) proteins, SMAC-mimetic (small-molecule second mitochondria-derived activator of caspases) with other therapeutic agents [32].

Some of the major challenges faced in the development of cancer-specific therapy are the unfavorable drug side effects and potential resistance gained by cancer cells. Therefore, there is a need for the recognition and discovery of such combinations of drugs that regulate independent pathways but work synergistically to effectively cause cancer cell death. One study demonstrated the combination of inhibitors of anti-apoptotic BCL2-like proteins and drugs that alter the balance of bioactive pro-apoptotic sphingolipids in a screen as a potential therapy to kill leukemia cells [33].

By altering the microenvironment and generating mutations, chemotherapy and other genotoxic drugs can also be used to increase immunogenicity and result in better immunotherapy outcomes [34]. One study reported increased susceptibility to systemic therapy with immunomodulatory antibodies in distant tumors as a result of localized therapy with oncolytic Newcastle disease virus (NDV), which induced inflammatory immune infiltrates [35].

Thus, establishing novel combinations of conventional chemotherapy with other therapeutic agents based on molecular information extracted from each tumor, to eradicate resistant leukemic blasts that persist in the hypoxic BM microenvironment, is an avenue that needs further exploration.

## Conclusions

AML is an aggressive and devastating disease that shows an initial response to chemotherapy but, if not eradicated in the first attempt, becomes increasingly resistant to treatment. The potential for eradicating AML lies in rational combinations of immunotherapies, the creation of a metabolically unfavorable microenvironment, and strategies to mitigate adaptive resistance. The growing immunotherapeutic resources markedly expand options but should be employed judiciously, cautiously, and in the right setting and careful consideration should be given to the sequence of immunotherapy.

Furthermore, the above-discussed approaches remain potentially susceptible to adaptive resistance by AML blasts using a variety of escape strategies that include the recruitment of immunosuppressive regulatory T cells or myeloid-derived suppressor cells, the creation of a metabolically unfavorable microenvironment, or the upregulation of inhibitory ligands. Thus, the potential for eradicating AML lies in rational combinations of immunotherapies with strategies to mitigate adaptive resistance by AML
